# Supplementary figures and images for: Lamin A/C-Dependent Translocation of Megakaryoblastic Leukemia-1 and β-Catenin in Cyclic Strain-Induced Osteogenesis
Source: Cells. 2021 Dec 14;10(12):3518. doi: 10.3390/cells10123518 (PMC8700688; doi:10.3390/cells10123518)

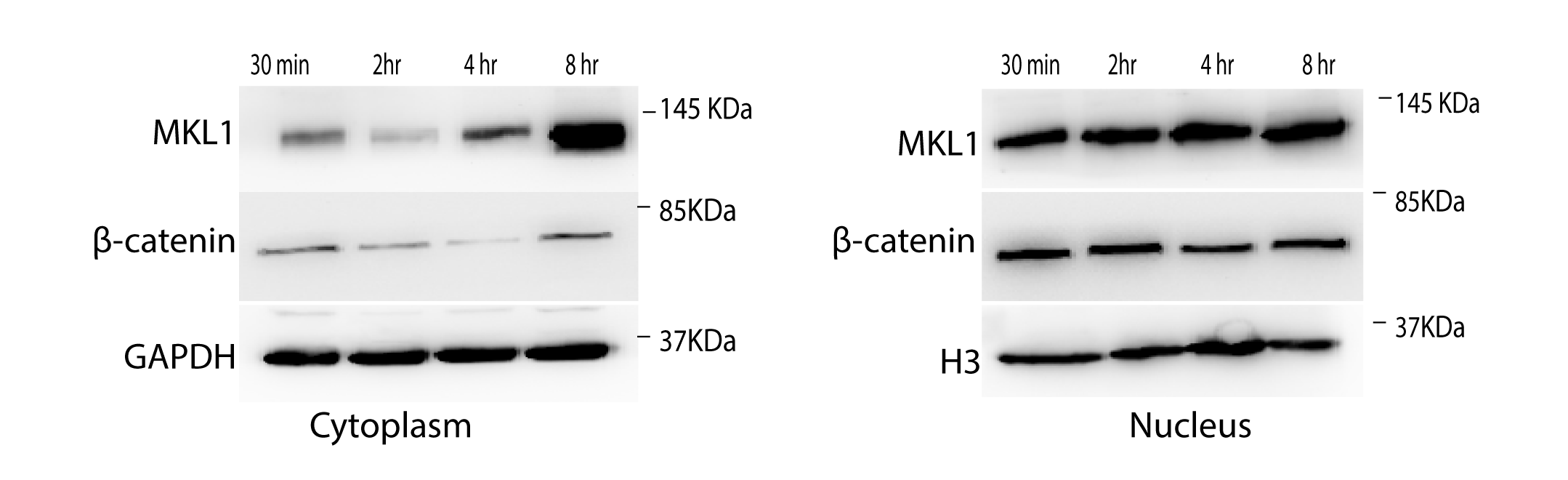

Supplement: Supplementary file 1 [file cells-10-03518-s001.zip › cells-1478063-supplementary/cells-1478063-supplementary.tif]
